# Herbal medicines for the treatment of cancer chemotherapy-induced side effects

**DOI:** 10.3389/fphar.2015.00014

**Published:** 2015-02-10

**Authors:** Shunsuke Ohnishi, Hiroshi Takeda

**Affiliations:** ^1^Department of Gastroenterology and Hepatology, Hokkaido University Graduate School of Medicine, Sapporo, Japan; ^2^Pathophysiology and Therapeutics, Faculty of Pharmaceutical Sciences, Hokkaido University, Sapporo, Japan

**Keywords:** Kampo, rikkunshito, hangeshashinto, goshajinkigan, chemotherapy-induced side effects

## Abstract

Accumulating evidence suggests that Japanese herbal medicines, called Kampo, have beneficial effects on cancer chemotherapy-induced side effects. Rikkunshito ameliorates cisplatin-induced anorexia through an antagonistic effect on the 5-HT receptors and by increasing the serum ghrelin levels. Hangeshashinto improves irinotecan-induced diarrhea and chemotherapy-induced mucositis by inhibiting the activity of β-glucuronidase as well as the synthesis of prostaglandin E2. Goshajinkigan prevents oxaliplatin-induced neurotoxicity, possibly through suppressing functional alterations of the transient receptor potential channels. In this review, we will summarize the currently available literature regarding the clinical efficacy and potential mechanisms of Kampo medicines in the treatment of cancer chemotherapy-induced side effects.

## INTRODUCTION

Cancer is a major public health problem in most developed countries; however, there have been notable improvements in the survival rate of patients over the past three decades owing to early detection and progress in medical treatment (DeSantis et al., 2014; Siegel et al., 2014). A substantial number of patients with cancer receive chemotherapy or chemoradiotherapy and benefit from treatment with anticancer drugs (DeSantis et al., 2014). However, because of their toxic effects on normal cells/tissues, anticancer drugs cause many side effects with a variety of symptoms, such as nausea, vomiting, anorexia, diarrhea, oral mucositis, and numbness. These side effects often compromise patients’ quality of life (QOL) and sometimes make it difficult to continue chemotherapy or chemoradiotherapy (Akin et al., 2010). Although many valuable strategies have been developed to treat or prevent these side effects, they are still insufficient (Gibson et al., 2013; McGuire et al., 2013; Hershman et al., 2014; Jordan et al., 2014). Therefore, an alternative or novel approach to treat or prevent these side effects is required.

Japanese traditional herbal medicines, called Kampo, were imported from China 1,500 years ago and developed independently (Motoo et al., 2011). Kampo medicines are currently prescribed by more than 80% of medical doctors in Japan and are covered by National Health Insurance. In recent years, several Kampo medicines have been investigated using animal models and clinical trials to assess their effects on chemotherapy-induced side effects. In this review, we describe the current status of several Kampo medicines in the treatment or prevention of chemotherapy-induced side effects as well as their underlying mechanisms.

## RIKKUNSHITO FOR THE TREATMENT OF CISPLATIN-INDUCED ANOREXIA

### CISPLATIN-INDUCED ANOREXIA

It is important to prevent and treat chemotherapy-induced side-effects such as anorexia, nausea, and vomiting to maintain patients’ QOL and to continue chemotherapy safely (Hesketh, 2008). Nausea and vomiting are caused by activation of vagal afferent neurons through the release of 5-HT and substance P from the enterochromaffin cells in the gut via 5-HT3 and neurokinin (NK)-1 receptors, respectively. Antagonists of 5-HT3 and NK-1 receptors have been developed and are widely used for the treatment of chemotherapy-induced nausea and vomiting (Roila et al., 2010; Basch et al., 2012). On the other hand, anorexia has been reported to be associated with other 5-HT receptors; 5-HT2R or 5-HT3R antagonists are associated with a decrease in food intake, leading to anorexia, as concluded from animal experiments (De Vry and Schreiber, 2000; Hayashi et al., 2005). 5-HT2CR is expressed mainly in the stomach (Wouters et al., 2007), whereas 5-HT2BR expression is restricted to the central nervous system (Giorgetti and Tecott, 2004).

International guidelines for antiemetic use have recommended antagonists for 5-HT3 and NK-1 receptors as well as corticosteroids for patients undergoing treatment with anticancer drugs including cisplatin (Roila et al., 2010; Basch et al., 2012). However, the effect of this treatment is still insufficient, because the complete response rate and complete control rate have been reported to be 40–75%, and anorexia as an adverse event was reported in approximately 15% of patients (Hesketh et al., 2003; Poli-Bigelli et al., 2003; Schmoll et al., 2006). In addition, food intake decreased to 25% of baseline by 7 days after administration of chemotherapy including cisplatin (Hiura et al., 2012b). These results suggest that gastrointestinal symptoms such as nausea, vomiting, and anorexia following chemotherapy persist for several days in many patients.

Ghrelin is a GH-releasing peptide isolated from the stomach as an endogenous ligand for GH secretagogue receptor 1a (GHS-R1a; Howard et al., 1996), a G-protein-coupled orphan receptor (Kojima et al., 1999). Ghrelin has 28 amino acids, with 3Ser acylated with an n-octanoyl residue, and this octanoylation is essential for the activation of ghrelin. Ghrelin is predominantly produced by the endocrine X/A-like cells of the stomach (Date et al., 2000) and plays various physiological roles as a circulating hormone, such as inducing GH release and food intake. More than 90% of circulating ghrelin is in an inactive form called desacylghrelin; however, recent studies have demonstrated that this desacylghrelin is also physiologically active (Baldanzi et al., 2002; Bedendi et al., 2003; Tsubota et al., 2005). Administration of ghrelin into the brain ventricles or veins of rodents induced food intake and weight gain (Nakazato et al., 2001). The hypothalamus has an arcuate nucleus that manages food intake, and certain food intake-stimulating neurons produce neuropeptide Y (NPY) and agouti-related protein (AgRP). The ghrelin receptor is expressed in the same neurons and is thus considered to stimulate food intake by activating the NPY/AgRP neurons (Nakazato et al., 2001). Ghrelin secreted from the stomach binds to the ghrelin receptor, which is produced in vagal afferent neurons and transported to the terminal of the afferent fiber, and inhibits electrical activity of the vagal afferent fiber. This signal is transmitted to the solitary nucleus in the medulla oblongata and then to the NPY and growth hormone releasing hormone (GHRH) neurons in the hypothalamus, leading to food intake and secretion of GH (Date et al., 2002). Recent studies have demonstrated that the blood level of ghrelin decreases after chemotherapy including cisplatin in animals as well as in humans (Takeda et al., 2008; Ohno et al., 2011; Hiura et al., 2012a). Therefore, ghrelin may be involved in cisplatin-induced anorexia.

### EFFECT OF RIKKUNSHITO ON CISPLATIN-INDUCED ANOREXIA

Rikkunshito is composed of 8 herbal medicines and is widely used in Japan to treat various gastrointestinal disorders (Tatsuta and Iishi, 1993; Yagi et al., 2004). Through animal experiments, we demonstrated the following: (1) cisplatin decreases serum ghrelin levels; (2) antagonists for the 5-HT2B or 5-HT2C receptors recover the decrease in serum ghrelin levels and food intake caused by cisplatin, respectively; (3) rikkunshito recovers the decrease in serum ghrelin levels and food intake caused by cisplatin; and (4) the flavonoids present in rikkunshito have an antagonistic effect on the 5-HT2B and 5-HT2C receptors (Takeda et al., 2008). Yakabi et al. (2010a,b) demonstrated that cisplatin decreases the expression of GHS-R1a and secretion of ghrelin in the hypothalamus via the 5-HT2C receptor, and rikkunshito ameliorates this effect. It has also been reported that rikkunshito antagonizes the 5-HT3 receptor (Tominaga et al., 2009).

Several clinical trials have been conducted to investigate the effect of rikkunshito on chemotherapy-induced anorexia. Ohno et al. (2011) performed a cross-over clinical trial using rikkunshito involving 10 patients with unresectable or recurrent gastric cancer treated with S-1 and cisplatin. They reported that rikkunshito attenuated the decrease in plasma acyl ghrelin levels, increased food intake during chemotherapy, and reduced the degree of anorexia caused by chemotherapy. Seike et al. (2011) evaluated the effect of rikkunshito in 19 patients with advanced esophageal cancer treated with docetaxel, 5-FU, and cisplatin. They reported that rikkunshito ameliorated chemotherapy-induced nausea and vomiting and improved the QOL score, particularly for mood and daily activity.

## HANGESHASHINTO FOR THE TREATMENT OF IRINOTECAN HYDROCHLORIDE (CPT-11)-INDUCED DIARRHEA

### CPT-11-INDUCED DIARRHEA

CPT-11 exerts an inhibitory effect on nucleic acid synthesis by inhibition of topoisomerase I and is widely used for the treatment of a variety of cancers (Rosen, 1998). One of the dose-limiting toxicities is late onset diarrhea (beginning more than 24 h after infusion) that leads to dehydration and electrolyte imbalance, making it a life-threatening condition (Abigerges et al., 1995). The underlying mechanism for CPT-11-induced late onset diarrhea involves direct damage to the intestinal mucosa induced by SN-38, a metabolite of CPT-11 (Hecht, 1998). CPT-11 is converted to SN-38, an active form of CPT-11 in the liver, and subsequently conjugates to inactive, non-toxic SN-38 glucuronide. Thereafter, SN-38 glucuronide is deglucuronidated to SN-38 by bacterial β-glucuronidase and induces mucosal damage and toxicity (Atsumi et al., 1991).

### EFFECT OF HANGESHASHINTO ON CPT-11-INDUCED DIARRHEA

Hangeshashinto is composed of seven herbs and is often used in Japan to treat diarrhea and acute gastroenteritis (Kase et al., 1997). Hangeshashinto is known to slow the enterohepatic circulation of SN-38. Baicalin in hangeshashinto has been shown to inhibit the activity of β-glucuronidase as well as the synthesis of prostaglandin E2 (Narita et al., 1993; Kase et al., 1997). In an animal experiment, hangeshashinto, and baicalin exhibited protective effects against intestinal toxicity caused by CPT-11 (Takasuna et al., 1995). A randomized controlled trial was performed involving 41 patients with non-small cell lung cancer treated with cisplatin and CPT-11, and the grade of diarrhea significantly improved after treatment with hangeshashinto (Mori et al., 2003). In addition, hangeshashinto significantly reduced the frequency of severe diarrhea (grade 3 or 4). Furthermore, the frequency of diarrhea was less than that reported previously (Komatsu et al., 2010). Only 1 out of 15 patients who received 125 mg/m^2^ of CPT-11 suffered from grade 3 diarrhea.

## HANGESHASHINTO FOR THE TREATMENT OF CHEMOTHERAPY-INDUCED ORAL MUCOSITIS

### CHEMOTHERAPY-INDUCED ORAL MUCOSITIS

Oral mucositis is a common complication of chemotherapy, affecting 18–40% of patients after the first chemotherapy cycle (Lopez-Castano et al., 2005). The morbidity of oral mucositis is primarily due to the pain associated with oral mucosal inflammation and ulceration, which affects food intake, oral hygiene, and QOL (Campos et al., 2014).

### EFFECT OF HANGESHASHINTO ON CHEMOTHERAPY-INDUCED ORAL MUCOSITIS

Hangeshashinto contains several prostaglandin E2-regulating ingredients (Kase et al., 1998; Kono et al., 2014). In a retrospective study with a small number of patients (*N* = 14), topical application of hangeshashinto improved oral mucositis (Kono, 2010). Aoyama et al. (2014) conducted a double-blind, placebo-controlled, randomized phase II study of hangeshashinto for oral mucositis induced by gastric cancer chemotherapy. Although hangeshashinto did not reduce the incidence of ≥2 oral mucositis, it tended to reduce the risk of oral mucositis in the patients who developed grade 1 oral mucositis. Yamashita et al. (2015) investigated the effect of hangeshashinto for chemoradiation-induced mucositis in head and neck cancer patients, and hangeshashito was associated with a significantly improved rate of completion of chemoradiation with cisplatin. In addition, serum albumin level was significantly maintained better in the hangeshashinto group than in the control group. Matsuda et al. (2013) are currently conducting a double-blind, placebo-controlled, randomized phase II study of hangeshashinto for oral mucositis induced by fluorinated pyrimidine-based colorectal cancer chemotherapy.

## GOSHAJINKIGAN FOR THE TREATMENT OF OXALIPLATIN-INDUCED NEUROTOXICITY

### OXALIPLATIN-INDUCED NEUROTOXICITY

Oxaliplatin, a platinum compound, is widely used for the treatment of various cancers, mainly colorectal cancer. However, oxaliplatin-induced neurotoxicity is the most common dose-limiting side effect (André et al., 2004; Haller et al., 2011). Although the underlying mechanism of oxaliplatin-induced neurotoxicity is not fully understood, it has been suggested that oxaliplatin accumulates in the dorsal root ganglia and produces axonal hyperexcitability and repetitive discharges due to changes in voltage-dependent sodium channels (Pasetto et al., 2006; Faber et al., 2012).

### EFFECT OF GOSHAJINKIGAN ON OXALIPLATIN-INDUCED NEUROTOXICITY

Goshajinkigan is composed of 10 herbal medicines and is widely used in Japan for the treatment of rhigosis, numbness or pain in the extremities, low back pain, and diabetic neuropathy (Tawata et al., 1994; Uno et al., 2005). Animal experiments have demonstrated that goshajinkigan prevents oxaliplatin-induced acute peripheral neuropathy without affecting its anti-tumor efficacy (Ushio et al., 2012) by suppressing functional alterations of the transient receptor potential (TRP) channels, particularly TRPA1 and TRPM8 (Kato et al., 2014; Mizuno et al., 2014). Retrospective studies and randomized controlled trials with a small number of patients have demonstrated that goshajinkigan significantly reduces the incidence of neurotoxicity, prolongs the duration of oxaliplatin treatment, and delays the time to the onset of neurotoxicity (Kono et al., 2011; Nishioka et al., 2011; Hosokawa et al., 2012). A placebo-controlled, double-blind randomized phase II study was conducted in patients with advanced or recurrent colorectal cancer treated with standard FOLFOX regimens; goshajinkigan appeared to have an acceptable safety margin and a promising effect in delaying the onset of grade ≥2 neurotoxicity without impairing FOLFOX efficacy (Kono et al., 2013). Yoshida et al. (2013) evaluated the efficacy of goshajinkigan for oxaliplatin-induced neuropathy in patients with colorectal cancer and demonstrated that goshajinkigan prevented exacerbation of neuropathy.

## CONCLUSION

Growing evidence suggests that Kampo medicines appear to have beneficial effects for the treatment or prevention of several chemotherapy-induced side effects. Because each Kampo medicine is not composed of a single substance, further basic studies should clarify which of the substances are responsible for the beneficial effects as well as the underlying mechanism of action. Although it is sometimes difficult to set appropriate and subjective endpoints for Kampo medicines, placebo-controlled, double-blind, randomized clinical trials similar to those for Western medicine will provide decisive evidence.

### Conflict of Interest Statement

The authors declare that the research was conducted in the absence of any commercial or financial relationships that could be construed as a potential conflict of interest. Hiroshi Takeda received grant support from Tsumura & Co. Shunsuke Ohnishi declares that the research was conducted in the absence of any commercial or financial relationships that could be construed as a potential conflict of interest.
